# The psychological mechanism of internet information processing for post-treatment evaluation

**DOI:** 10.1016/j.heliyon.2022.e09351

**Published:** 2022-04-28

**Authors:** Quan-Hoang Vuong, Tam-Tri Le, Viet-Phuong La, Minh-Hoang Nguyen

**Affiliations:** Centre for Interdisciplinary Social Research, Phenikaa University, Yen Nghia Ward, Ha Dong District, Hanoi, 100803, Viet Nam

**Keywords:** Digital healthcare, Healthcare quality, Public communication, Mindsponge mechanism, Internet information

## Abstract

Digital healthcare has been greatly benefiting the public health system, especially during the COVID-19 pandemic. In digital healthcare, information communication through the Internet is crucial. The current study explores how patients' accessibility and trust in Internet information influence their decisions and *ex-post* assessment of healthcare providers by employing the Bayesian Mindsponge Framework (BMF) on a dataset of 1,459 Vietnamese patients. We find that patients’ accessibility to Internet information positively affects the perceived sufficiency of information for choosing a healthcare provider, and their trust in the information intensifies this effect. Internet information accessibility is negatively associated with post-treatment assessment of healthcare providers, and trust also moderates this effect. Moreover, patients considering professional reputation important while making a decision are more likely to regard their choices as optimal, whereas patients considering services important have contradicting tendencies. Based on these findings, a concern about the risk of eroding trust toward Internet sources about healthcare information is raised. Thus, quality control and public trust-building measures need to be taken to improve the effectiveness of healthcare-related communication through the Internet and facilitate the implementation of digital healthcare.

## 1 Introduction

During the combat against the COVID-19 pandemic, digital healthcare has played an important role in the public health system. Due to the rapid changes in demand, capacity, and conditions of healthcare services attributable to the COVID-19 pandemic, the conventional face-to-face model must be reconfigured. Many countries, therefore, have incorporated digital technology into their new healthcare models for improving efficiency, reducing transmission risks, and enhancing flexibility (Gunasekeran et al., 2021). Integrating digital technology into healthcare systems is also essential among low- and middle-income countries (LMICs). It is one of the non-pharmaceutical interventions that help minimize the adverse impacts of the COVID-19 pandemic (Chowdhury et al., 2020; Walker et al., 2020). Mitgang et al. (2021) suggest that LMICs’ healthcare systems can be markedly improved by employing information and communication technologies (ICTs) to enhance direct communication with the public, develop scale-proven and innovative service delivery models, and empower the frontlines. Understanding the framework for information management is crucial in a global public health crisis (Vuong et al., 2022), especially considering potential negative public perceptions toward science (Vuong, 2018).

Digital health, which was first introduced by Frank (2000), provides consumers with five fundamental functions: 1) information dissemination, 2) informed decision-making support, 3) health promotion, 4) a medium to exchange information and support, and 5) self-care improvement and demand management. Even though the field has rapidly expanded to cover other scientific concepts and technologies, like artificial intelligence, analytics, mobile applications, telemedicine, etc. (Mathews et al., 2019), dissemination and communication of information through the Internet still play crucial roles in the modern digital health system, and even digital health ecosystem (Serbanati et al., 2011). Regarding the relationship between human health and digital technology, artificial intelligence is becoming more and more helpful in supporting medical data processing (Holzinger et al., 2022); for example, the European Commission has clear guidance on the legal use of artificial intelligence in medical treatments (Stöger et al., 2021). While artificial intelligence is important for building a sustainable modern medical system, the issues of ethics and public trust require careful consideration as well as further technological advancement (Muller et al., 2021).

The Internet is a useful source of information for various health issues, disease prevention, treatment methods, psychological stress, etc. (Galarce et al., 2011). Dickerson et al. (2011) find that men with cancer seek information about disease and treatments from the Internet for decision making, organizing information, and navigation. Online health information has a mediation effect on the association between social support and healthy eating intentions (McKinley and Wright, 2014). The Internet is also a channel for acquiring healthcare reform information (Thompson et al., 2012). As Internet information-seeking behaviors increasingly contribute to people's health-related decisions, their behavioral outcomes are also altered accordingly. A study on Chinese patients suggests that the quality and source of internet-based information positively influence patient compliance, which improves treatment effectiveness (Lu et al., 2018). Furthermore, trust in healthcare providers, online health information, and Internet use significantly predict the intention to discuss health with their providers (Hong, 2008).

Healthcare information is a crucial factor in consumer-driven healthcare models (Harris et al., 2008). Quality information can help improve quality and minimize healthcare costs by empowering patients and improving their informed decision-making in choosing healthcare providers (Cable.co.uk, 2004; Rains, 2007). Thanks to the rapid development of ICTs, the Internet has become increasingly available. It is now one of the trusted information sources about care providers, apart from mass media, professional experts, and family members and friends (Harris et al., 2008; Rains, 2007; Tu and Lauer, 2008). According to Lemire et al. (2008), the use of the Internet as a preferred source of information is associated with five factors: perceived usefulness, trust in the information, opinions of health professionals, the reporting media, and personal health concerns. Despite the mounting literature regarding the effects of internet-based information on multiple health-related information, little is known about the relationship between the psychological process involving online information and patients' selection and post-treatment evaluation of healthcare providers. Information from the Internet might be both good and bad (Tonsaker et al., 2014). However, no matter how it is, if the information is used to decide on a healthcare provider, it might affect patients' post-treatment evaluation. Thus, good evaluation of decisions based on Internet information is expected to increase perceived usefulness and trust toward the information sources, and vice versa, and eventually influence patients’ information-seeking behaviors in the future (Lemire et al., 2008; Sheng and Simpson, 2015).

Vietnam – one of the LMICs with more than 96 million population – has been heavily affected by the recent outbreaks despite the successful containment during early COVID-19 waves. Since July 1, 2021, the total of COVID-19 infected cases in Vietnam increased by about 30 times (from 17,727 to 550,996 cases) in less than three months (until September 7, 2021) (Ritchie et al., 2020). The devastating situation is attributable to a combination of factors, such as the high transmissibility of the Delta variant, slow vaccine rollout, etc. Inadequate development of the digital health system is also one of the significant contributors to the outbreak (Bui et al., 2021). However, Vietnam has great potential to capitalize on the Internet for communicating health and healthcare information with patients. Approximately 70% of the Vietnamese population has access to the Internet, and Vietnam has the lowest price of Internet services in South East Asia (Cable.co.uk, 2004; The World Bank, 2020). Bui et al. (2021) stipulate that the inadequacy results from supply-side problems, such as the lack of strong governance, infrastructure, and staff capability for digital health development and deployment. Besides, the challenges of digital health can also be derived from societal factors (e.g., low public acceptance, medical misinformation dissemination on the Internet, unequal accessibility towards digital services, etc.) (Cummins and Schuller, 2020; Lennon et al., 2017).

Therefore, understanding the patients' psychological process of Internet information for selection and *ex-post* evaluation of healthcare providers can provide useful insights that support developing countries aiming to develop a better digital healthcare system, like Vietnam. According to the mindsponge information processing mechanism (Vuong and Napier, 2015), perceived information accessibility and trust are two fundamental elements of an individual's psychological process. The current study aims to examine the following two research questions using the Bayesian Mindsponge Framework (BMF) – the combination of the mindsponge mechanism and Bayesian analysis (Nguyen et al., 2021). A detailed explanation of the theoretical foundation based on BMF will be presented in the Model Construction subsection.

1. How do accessibility and trust in Internet information influence patients' decision of choosing healthcare providers?
2. How do accessibility and trust in Internet information influence patients' post-treatment evaluation of healthcare providers?

## 2 Methodology

### 2.1 Materials

The current study employed the dataset of 1459 patients visiting 30 hospitals across Northern Vietnam. The data were collected by face-to-face interviews conducted by a six-member data team from the fourth quarter of 2015 to the beginning of 2016. The full dataset and its descriptor are available in *Data in Brief* and can be retrieved from the following URL: https://linkinghub.elsevier.com/retrieve/pii/S2352340916302803. The survey collection was designed and implemented by Hanoi-based Vuong & Associates, with the ethical standards maintained by the institutional regulation and decision, numbered V&A/15#1, dated October 19, 2015. The team members were carefully instructed with written rules and standards of research ethics. Respondents were also asked to accept written consent before participating in the survey.

Among 1459 participants, more than half were female patients (64.56%), while male patients constituted 35.57%. Their average age was approximately 32. The proportion of non-poor patients (78.96%) was almost four times higher than poor patients (21.04%). The majority of patients resided in urban areas (73.06%), while the rest came from rural (22.62%) or remote areas (4.32%).

Seven variables were retrieved from the dataset for performing Bayesian analysis. Most of them are binary variables; only the patients’ perceived accessibility to Internet information is represented by a numerical variable (*Internet*). Table 1 shows seven variables as well as their detailed description and how they were coded. *Sufficiency* and *Posttreatment* are two outcome variables, whereas the other five variables are predictor variables.

**Table 1:** Variables’ detailed description.

Name	Variable	Data type	Description
Perceived sufficiency of the information	*Sufficiency*	Binary	Patients' subjective assessment of information sufficiency for choosing a healthcare provider. ‘Sufficient’ was coded as 1, ‘Not sufficient’ as 0.
Post-treatment assessment	*Posttreatment*	Binary	Patients' post-treatment assessment of whether a patient's choice was the best available. ‘Optimal’ was coded as 1, ‘Not optimal’ as 0.
Accessibility of Internet information	*Internet*	Numerical	Patients' perceived accessibility to information related to the healthcare provider on the Internet. ‘Limited and difficult’ was coded as 1, ‘Somewhat limited but still available’ as 2, and ‘Easy and convenient’ as 3.
Trust towards Internet information	*Internet_Trust*	Binary	Patients' trust towards the information related to the healthcare provider on the Internet. ‘Believe’ is coded as 1, ‘Only for reference when needed’ as 0.
Importance of provider's services	*Services*	Binary	Patients' perceived importance of provider's services in the determination of healthcare provider. ‘Decisive’ is coded as 1, ‘Indecisive’ as 0.
Importance of professional reputation	*Reputation*	Binary	Patients' perceived importance of a provider's reputation in the determination of healthcare provider. ‘Decisive’ is coded as 1, and ‘Indecisive’ is coded as 0.
Importance of provider's cost	*Cost*	Binary	Patients' perceived importance of provider's cost in the determination of healthcare provider. ‘Decisive’ is coded as 1, ‘Indecisive’ as 0.

### 2.2 Model construction

The present investigation employs the BMF, also known as the Bayesian Mindsponge analytical approach, to study patients’ psychological mechanisms of Internet information processing for selecting and evaluating healthcare providers. The mindsponge information processing framework (Vuong and Napier, 2015) was used as the theoretical foundation to construct models, while Bayesian analysis was used to explore the constructed models statistically. BMF, proposed by Nguyen et al. (2021), has been shown to effectively examine the underlying psychological mechanisms of behaviors and attitudes (Vuong, 2022; Vuong et al., 2021a, 2021b).

The mindsponge mechanism demonstrates how the mind receives and filters information, accepts or rejects values, and updates itself in the process. Information accessibility and favorable evaluation of the information are two fundamental conditions for a new piece of information to be accepted into the mindset. Regarding the first condition, this requires both objective availability and perceived accessibility of the information. The information needs to exist, be reachable, and be considered reachable to be received by the mind. Regarding the second condition, when the information is received, it has to go through the multi-filtering system, consisting of many cost-benefit judgments based on related information gathered from the environment and references of existing trusted values from the mindset (formerly accepted values). Suppose the total perceived benefit of the information's value is greater than its perceived cost. In that case, it will be accepted into the mindset and becomes a new trusted value, and vice versa (rejection).

**Figure 1:**
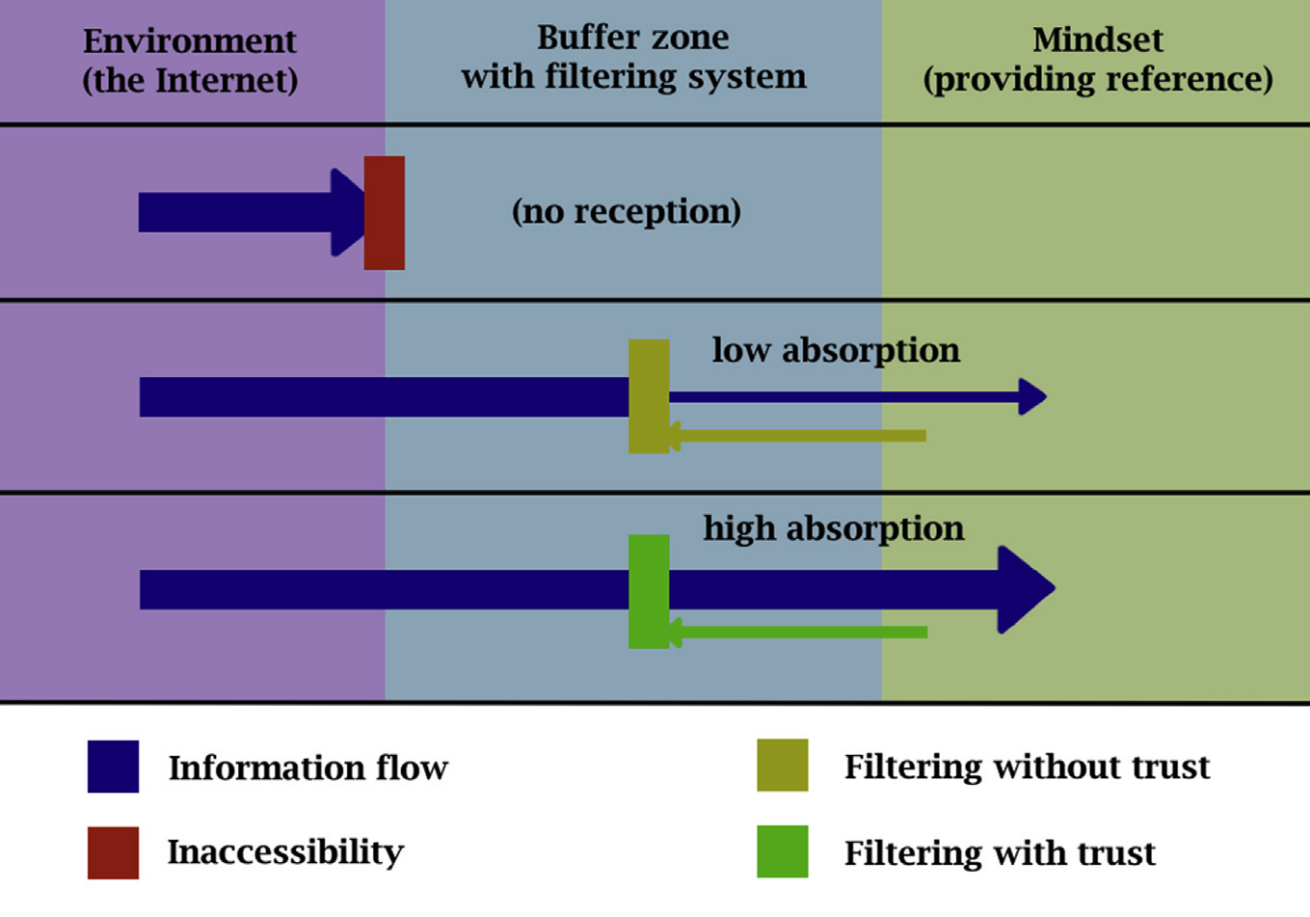
Psychological mechanism of Internet information processing.

Along the mindsponge process, trust (or “trust guard”) has a particularly important role. Trust is a special reference from the mindset based on formerly accepted values (or information) to justify the certainty or uncertainty of the information. Thus, it can greatly influence the cost-benefit judgments, which help speed up the evaluation process. Normally, trust (or distrust) is applied to a source of information or a group of information-carrying certain similar properties. By doing so, the mind can save time and energy by quickly accepting or discarding information belonging to the same source or group without conducting the whole evaluation process again for each value. This natural trust mechanism has advantages (e.g., overall efficiency to make a decision) and disadvantages (e.g., lack of consideration of inaccurate and fake information). It should be noted that the mindsponge process, just like a human, has a feedback loop, so individuals’ trust and cost-benefit judgments can be changed if their core values in the mindset are replaced. The psychological process of Internet information is visualized in Figure 1.

Based on the theoretical foundation briefly presented above, we begin by assuming that a patient needs a certain amount of information to make a decision. Thus, the accessibility to Internet information will positively influence the patient's perceived sufficiency of information to choosing a provider. Such influence will be facilitated by the patient's level of trust towards the information: more trust in the information means easier reception of that information into the mindset. Hence, Model 1 is proposed to test our first hypothesis:

Model1:Sufficiency~α+Internet+Internet∗Internet_Trust

If the associations in Model 1 are validated, we continue with Model 2; otherwise, the analysis will be stopped. The patients made a choice based on the information absorbed into their mindsets, including Internet and non-Internet information. Thus, their *ex-post* evaluation of the treatment would be based on comparing three types of information: formerly collected information (internet and non-Internet information) and information from real experience. Thus, we assume that accessibility and trust towards Internet information will influence the *ex-post* evaluation of whether the selected healthcare provider is optimal or not. Model 2 can be presented as follows:

Model2:Posttreatment~α+Internet+Internet∗Internet_Trust

Next, we constructed Model 3 by incorporating three aspects that might influence the post-treatment evaluation among patients: cost, professional reputation, and services. There are two reasons for doing so. First, adding more variables to the model will help validate the robustness of Model 2's findings. If the effects of accessibility and trust towards Internet information remain robust, they can be deemed reliable. More details of the validation will be explained in the following subsection. Model 3 also provides insights into the associations of post-treatment evaluation with the importance of cost, professional reputation, and services in selecting providers, which helps suggest issues that can be improved upon while disseminating information. Combining all parameters, we derive the following model:

Model3:Posttreatment~α+Internet+Internet∗Internet_Trust+Cost+Reputation+Services

### 2.3 Method and validation

We employed Bayesian analysis with the Markov Chain Monte Carlo (MCMC) technique in the present study for several reasons. Firstly, Bayesian inference treats all parameters (including unknown ones) as probabilities, which helps avoid the pitfall of *p*-value over-dependence that leads to the current reproducibility crisis in social sciences and especially psychology (Baker, 2015; Open Science Collaboration, 2015). Secondly, the properties of Bayesian inference are suitable for the explanatory research design of the present study, which examines the psychological process of receiving and filtering information for choosing a healthcare provider and evaluating the choice after treatment. With all parameters treated probabilistically, Bayesian analysis helps us consider the influence of other unknown factors while upholding the law of parsimony (Csilléry et al., 2010). Thirdly, the Markov Chain Monte Carlo technique can generate a large set of parameters’ iterative samples through stochastic processes of Markov chains, which provides a sufficient sample size for fitting complex models, including nonlinear relationships (Kerkhoff and Nussbeck, 2019).

A four-pronged validation strategy was employed to validate the simulated posterior results. Initially, we checked the model's goodness-of-fit using the Pareto smoothed importance-sampling leave-one-out cross-validation (PSIS-LOO) strategy (Vehtari et al., 2017). If all *k* values shown on the PSIS diagnostic plot are lower than 0.5, the model can be deemed acceptable (not under-fit nor over-fit). In the second step, we examined the Markov chain central limit theorem, which assumes that iterative samples in a Markov chain are independent (or not convergent). The effective sample size (n_eff) and Gelman shrink value (Rhat), as well as the trace plot, Gelman plot, and autocorrelation plot, were employed to diagnose the convergence.

Although the prior distributions are assumed to be “uninformative” to minimize the subjective influence on the simulated results, we also performed the “prior-tweaking” technique to check the simulated results’ robustness. In particular, we reran the analysis using distinct prior distributions (demonstrating our belief and disbelief toward the acquired results) of a parameter. If the simulated results only slightly change, the findings can be considered robust. Finally, we inserted additional variables into Model 2, creating Model 3 for robustness validation. If the associations of accessibility and trust towards Internet information with the post-treatment evaluation in Model 3 are not subject to change, the findings will be validated.

The Bayesian analysis was conducted using the **bayesvl** R package due to several advantages: i) being an open program, ii) having good visualization power, and iii) supporting the transparent operation (Vuong et al., 2020). The data and codes utilized for all statistical analyses and result presentations are available in the following repository: https://osf.io/9ukwg/.

## 3 Results

Out of 1459 patients, 31.53% assessed their treatment choices as being optimal, while 46.40% perceived that they had sufficient information to make good decisions. Regarding Internet information, most of the patients (41.95%) said that Internet information access was easy and convenient. Half of the patients (49.76%) believed in the Internet information, whereas the rest only used Internet information as a reference when needed.

### 3.1 Model 1: psychological process of perceived sufficiency

**Figure 2:**
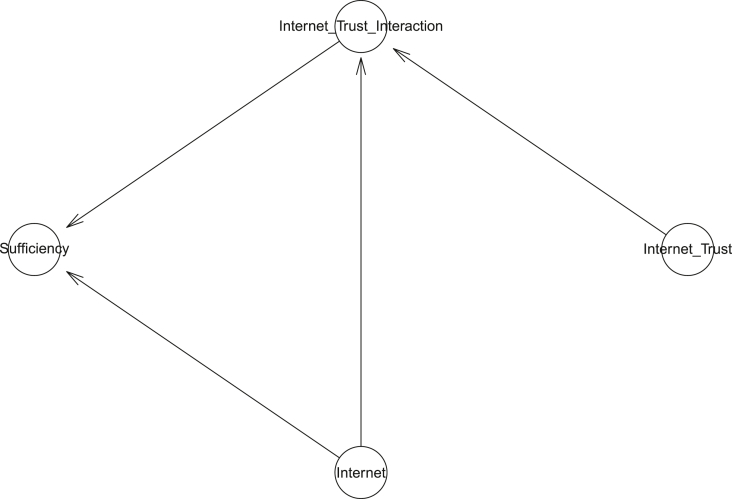
Model 1's logical network.

The first model investigated the effects of accessibility and trust towards Internet information on the perceived sufficiency of information to make a good choice of healthcare provider. Figure 2 demonstrates Model 1's logical network.

**Figure 3:**
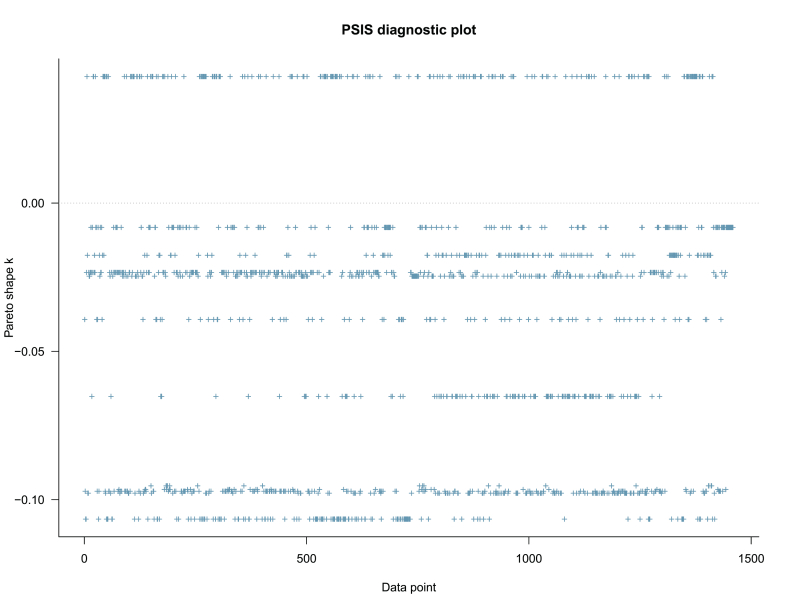
Model 1's PSIS diagnostic plot.

The model was first validated using the PSIS diagnostic plot shown in Figure 3. All the *k* values are below 0.5, which indicates the model's high goodness-of-fit with the data.

**Figure 4:**
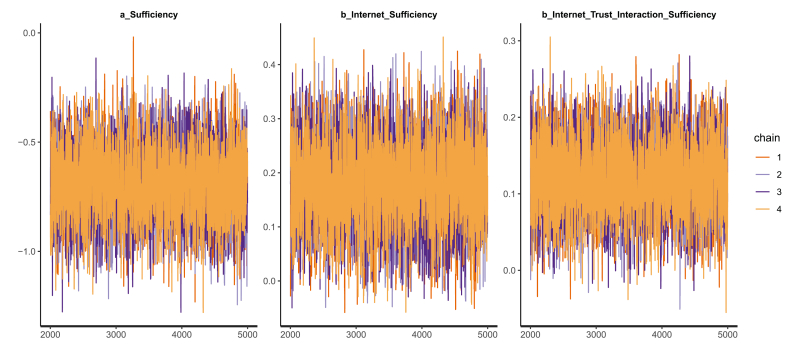
Model 1's trace plots.

**Figure 5:**
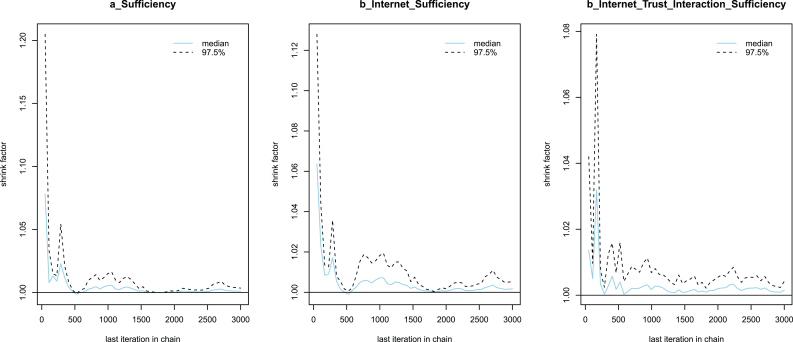
Model 1's Gelman plots.

**Figure 6:**
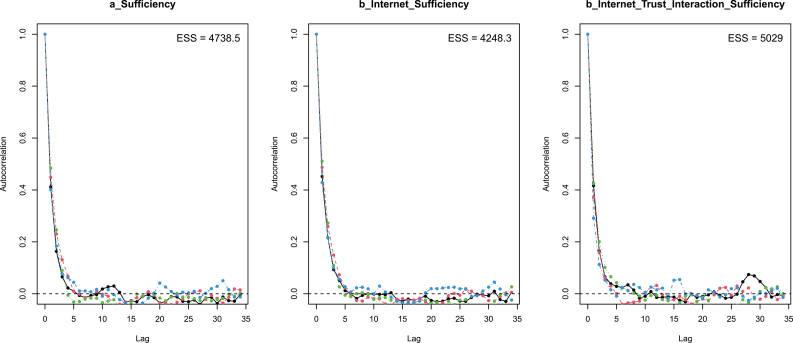
Model 1's autocorrelation plots.

The posterior coefficients' Markov chains can be deemed convergent as their n_eff values are larger than 1,000 and Rhat values are equal to 1 (see Table 2). The convergence is visually validated by the “healthy” Markov chains presented in the trace plots: good-mixing and stationary (see Figure 4). In the Gelman plots, the shrink factors drop rapidly to 1 (see Figure 5); the autocorrelation plots imply a substantial decline of autocorrelation levels after a certain number of lags (see Figure 6). These signals suggest that the Markov chains are well-convergent, and thus the central limit theorem is held in Model 1's simulation.

**Table 2:** Model 1's simulated posteriors.

Parameters	Uninformative prior		Prior-tweaking (belief on effect)		Prior-tweaking (disbelief on effect)		n_eff∗	Rhat∗
	Mean	SD	Mean	SD	Mean	SD		
*Constant*	-0.69	0.16	-0.68	0.16	-0.69	0.16	4691	1
*Internet*	0.18	0.08	0.17	0.08	0.18	0.08	4312	1
*Internet∗Internet_Trust*	0.12	0.05	0.13	0.05	0.12	0.05	5021	1

∗n_eff and Rhat values presented in the tables are the effective sample sizes and Gelman values taken from the simulated results using uninformative prior.

**Figure 7:**
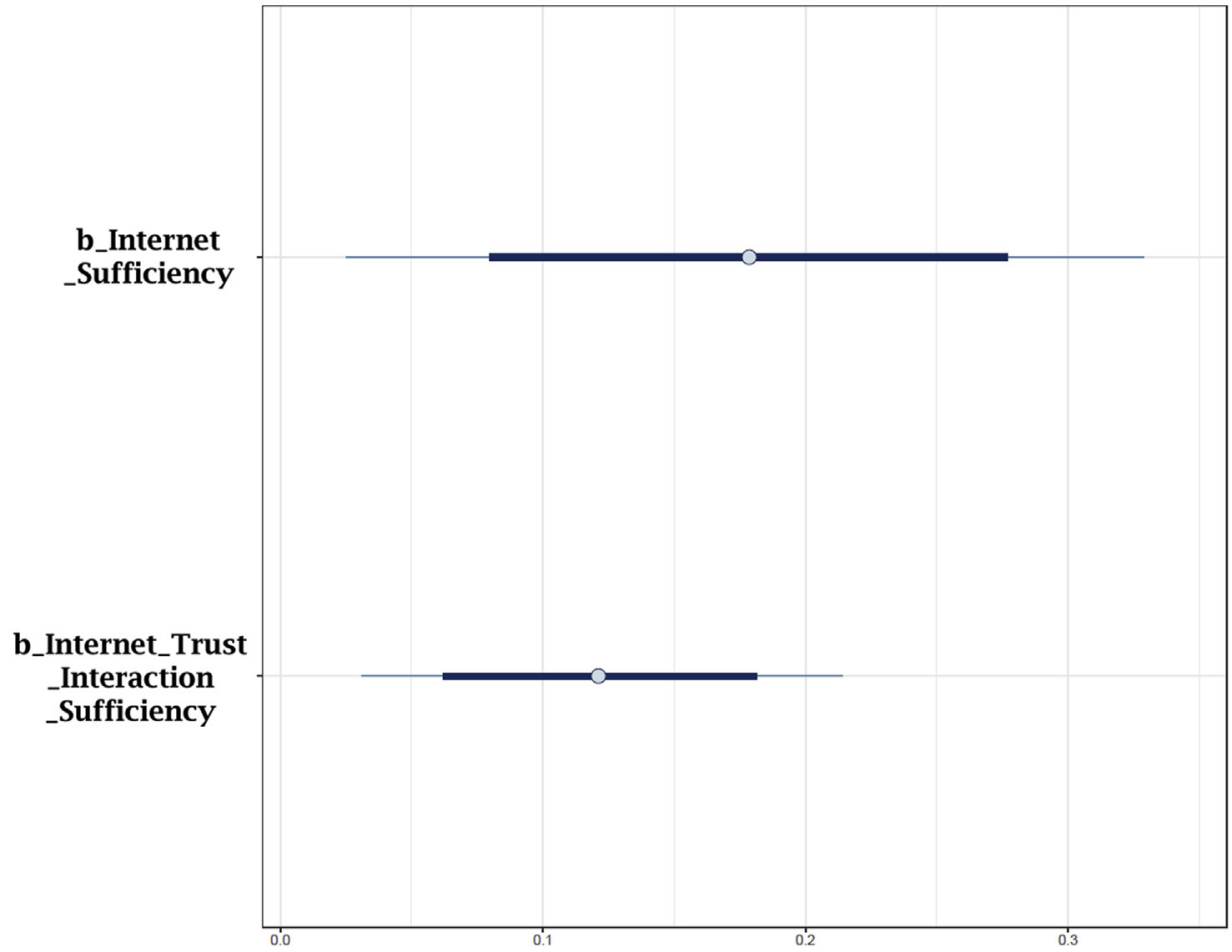
Distributions of Model 1's posterior coefficients on an interval plot.

As can be seen in Table 2, accessibility to Internet information is positively associated with the perceived sufficiency of information for choosing a healthcare provider ($\pi_{Internet}$ = 0.18 and $\sigma_{Internet}$ = 0.08). The effect of accessibility to Internet information is also intensified by the patient's trust ($\pi_{Internet*Internet\_Trust}$ = 0.12 and $\sigma_{Internet*Internet\_Trust}$ = 0.05). In other words, the effect of accessibility to Internet information on perceived information sufficiency is stronger among patients trusting the Internet data than among those who do not. The positive effects of accessibility and trust towards Internet information simulated based on Model 1 are highly reliable as the coefficients' probability distributions are located entirely on the positive side of the *x*-axis (see Figure 7).

To validate the simulated results, we performed the prior-tweaking technique on *Internet∗Internet_Trust* using two prior distributions: belief on effect and disbelief on effect. The prior demonstrating our belief that the moderation effect of *Internet_Trust* is positive (belief on effect) was set using a normal distribution with the mean being 0.5 and standard deviation being 0.3. In contrast, a prior demonstrating our disbelief that there is a moderation effect of *Internet_Trust* (disbelief on effect) was set using a normal distribution with the mean being 0 and standard deviation being 0.3. The simulated results using priors demonstrating belief and disbelief on the effect of *Internet_Trust* remain almost similar to the generated results employing uninformative priors (see Table 2). This outcome highlights the model's high resistance to initial changes of priors; in other words, the model is robust.

### 3.2 Model 2: psychological process of post-treatment evaluation

**Figure 8:**
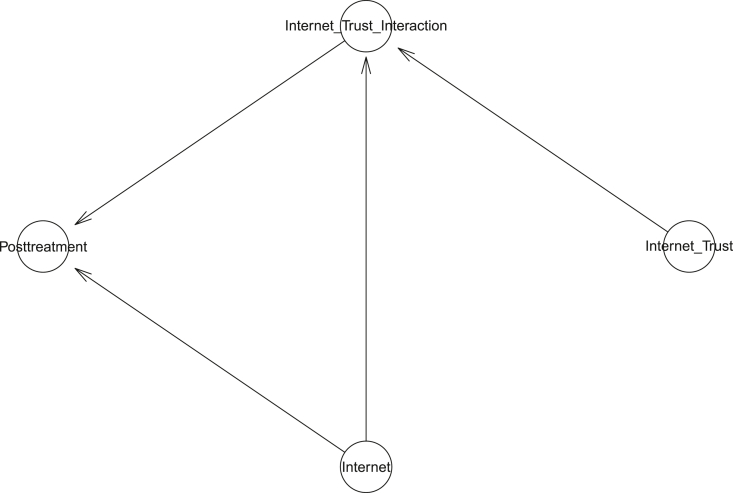
Model 2's logical network.

Given the effects of accessibility and trust towards Internet information on the perceived information for decision making, we continued with the second model, which examines how patients' accessibility and trust towards Internet information influence the *ex-post* evaluation. Model 2's logical network can be visualized in Figure 8.

**Figure 9:**
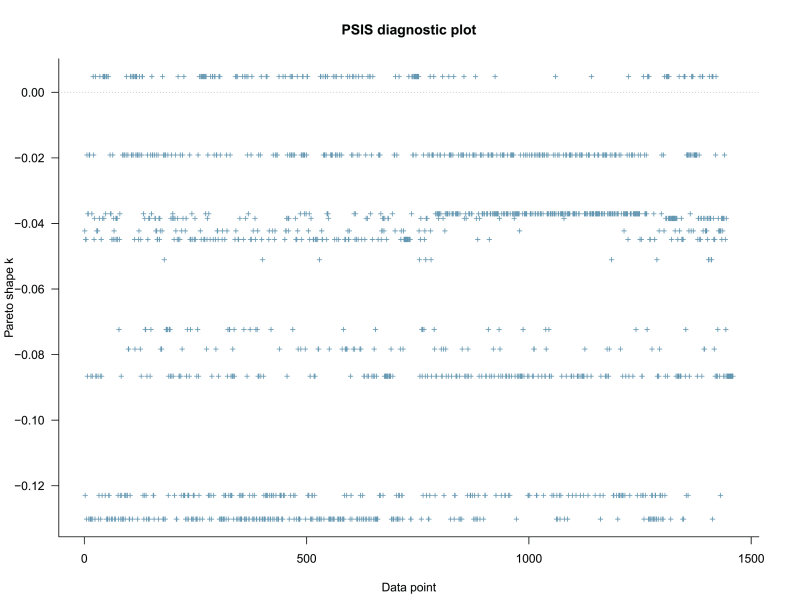
Model 2's PSIS diagnostic plot.

Model 2 is well-fitted with the data as all *k* values shown in the PSIS diagnostic plot are lower than 0.5 (see Figure 9).

**Figure 10:**
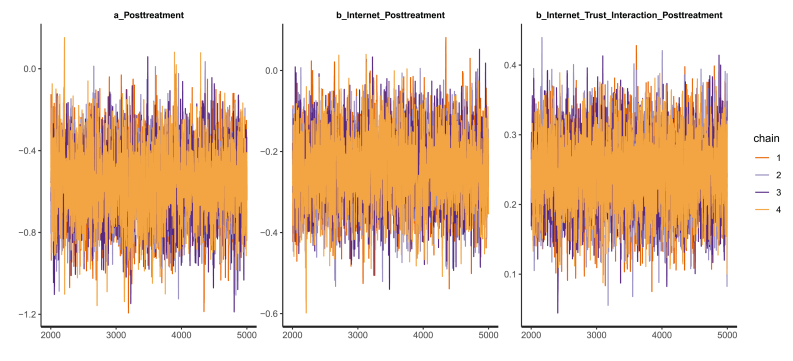
Model 2's trace plots.

**Figure 11:**
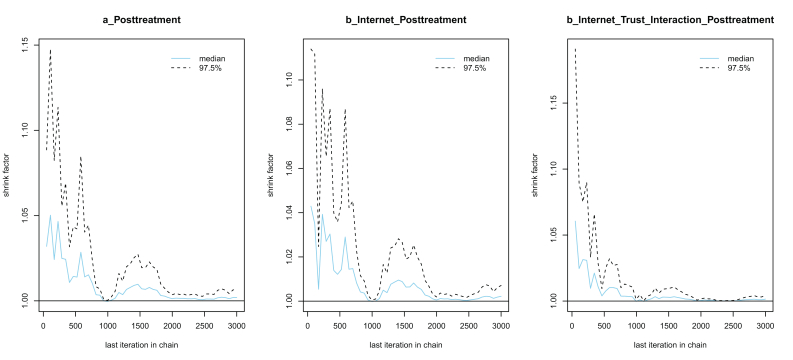
Model 2's Gelman plots.

**Figure 12:**
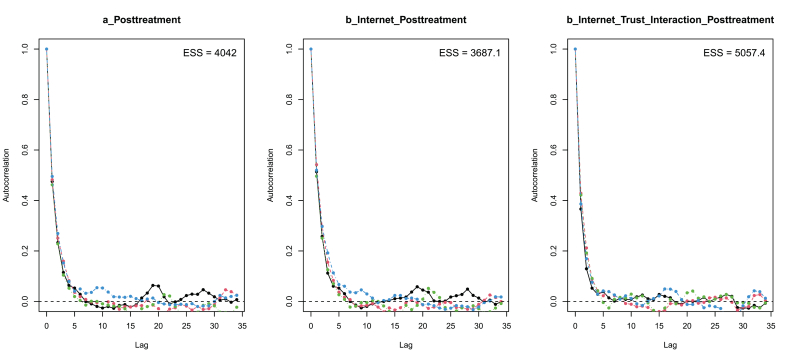
Model 2's autocorrelation plots.

The convergence diagnostic statistics of simulated posteriors show that all parameters’ Markov chains are well-convergent. Specifically, n_eff values are larger than 1000, and Rhat values are equal to 1. The model convergence is confirmed by the well-mixed Markov chains in the trace plots (see Figure 10) and the rapid declines of shrink factors and autocorrelation levels in Gelman and autocorrelation plots, respectively (see Figures 11 and 12, respectively).

**Figure 13:**
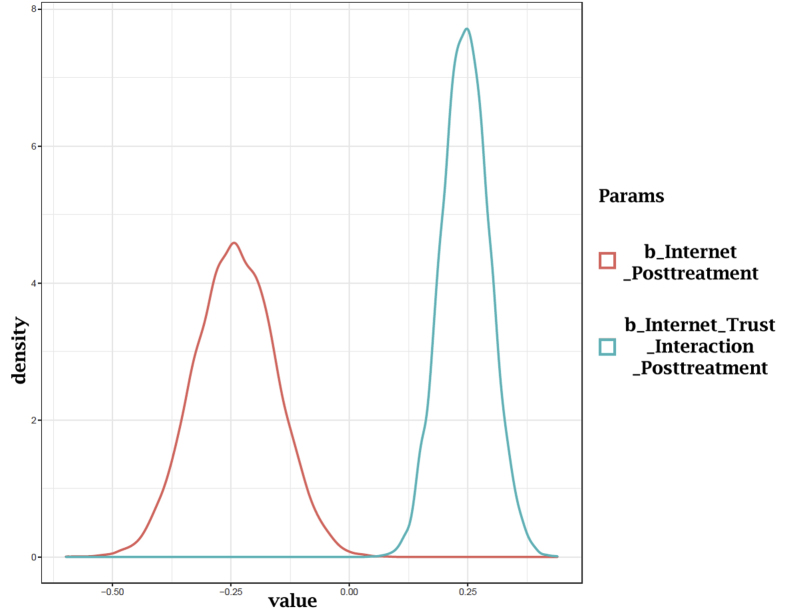
Distributions of Model 2's posterior coefficients on a density plot.

It is found that the accessibility to Internet information is negatively associated with the *ex-post* evaluation of provider choice ($\pi_{Internet}$ = -0.24 and $\sigma_{Internet}$ = 0.09). Nonetheless, patients' trust in Internet information negates the negative effect of accessibility to Internet information on the evaluation ($\pi_{Internet*Internet\_Trust}$ = 0.24 and $\sigma_{Internet*Internet\_Trust}$ = 0.05). The probability distributions of Model 2's coefficients are illustrated in Figure 13. The distribution of *Internet* lies mostly on the negative side. In contrast, the distribution of *Internet∗Internet_Trust* lies entirely on the positive side (see Figure 13), so the effect of *Internet* and the moderation effect of *Internet_Trust* can be deemed reliable. Applying the same tweaking technique to the prior distribution of *Internet∗Internet_Trust*, we also obtained results independent of initial prior modification (see Table 3), which implies the robustness of Model 2.

**Table 3:** Model 2's simulated posteriors.

Parameters	Uninformative prior		Prior-tweaking (belief on effect)		Prior-tweaking (disbelief on effect)		n_eff∗	Rhat∗
	Mean	SD	Mean	SD	Mean	SD		
*Constant*	-0.56	0.17	-0.55	0.17	-0.56	0.17	4021	1
*Internet*	-0.24	0.09	-0.25	0.09	-0.24	0.09	4038	1
*Internet∗Internet_Trust*	0.24	0.05	0.25	0.05	0.24	0.05	4623	1

∗n_eff and Rhat values presented in the tables are the effective sample sizes and Gelman values taken from the simulated results using uninformative prior.

### 3.3 Model 3: robustness check

**Figure 14:**
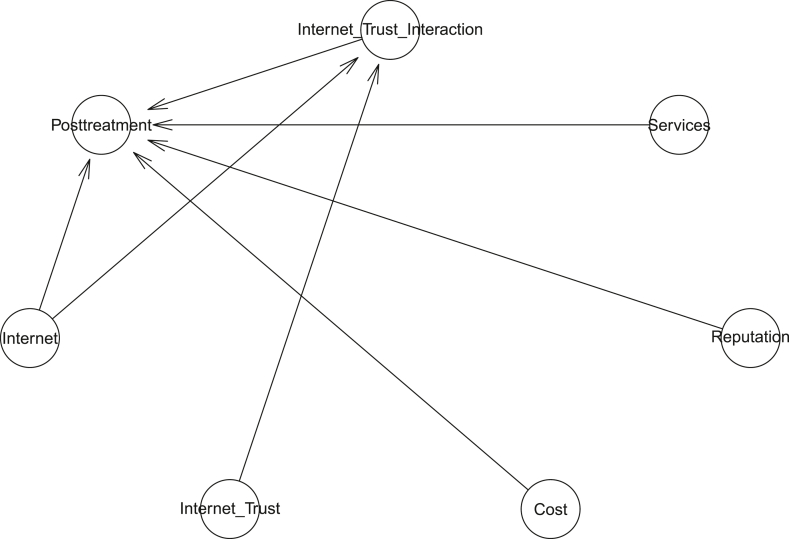
Model 3's logical network.

Finally, we constructed Model 3 by incorporating *Cost*, *Reputation*, and *Services* into Model 2 to check the robustness of Model 2's results. Model 3's logical network is illustrated in Figure 14.

**Figure 15:**
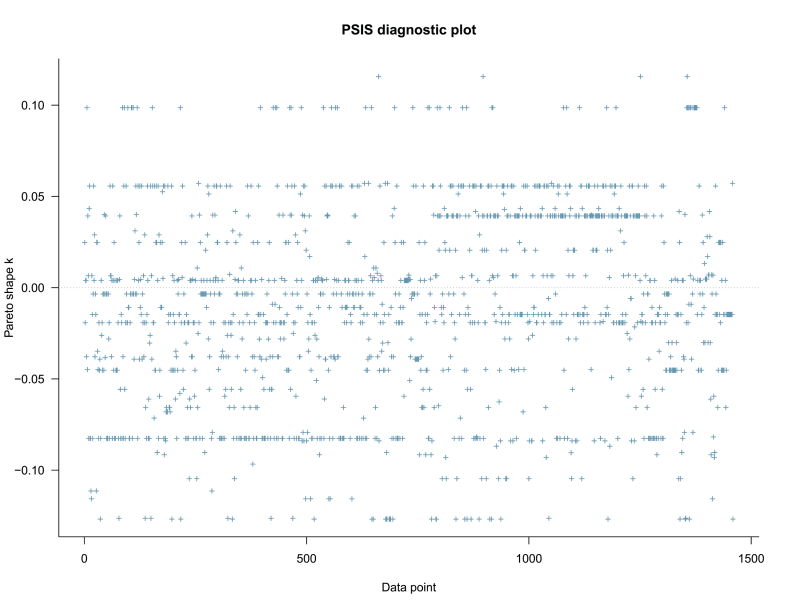
Model 3's PSIS diagnostic plot.

Despite adding more variables into the model, the PSIS-LOO test still shows that the model is not over-fit; Figure 15 stipulates that all *k*-values are below 0.5.

**Figure 16:**
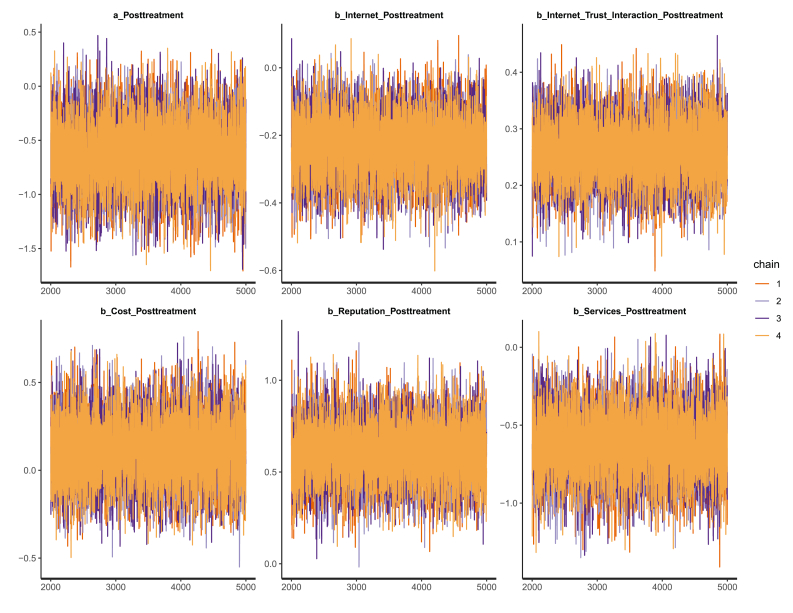
Model 3's trace plots.

**Figure 17:**
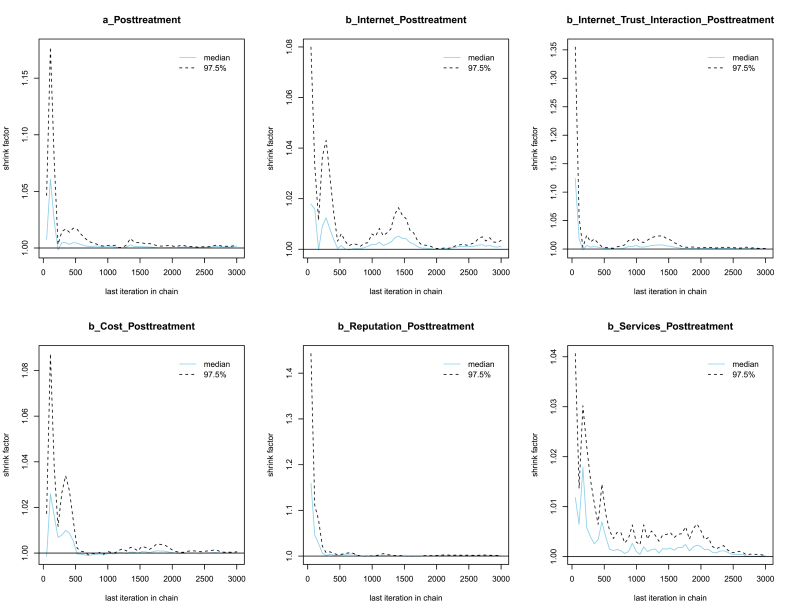
Model 3's Gelman plots.

**Figure 18:**
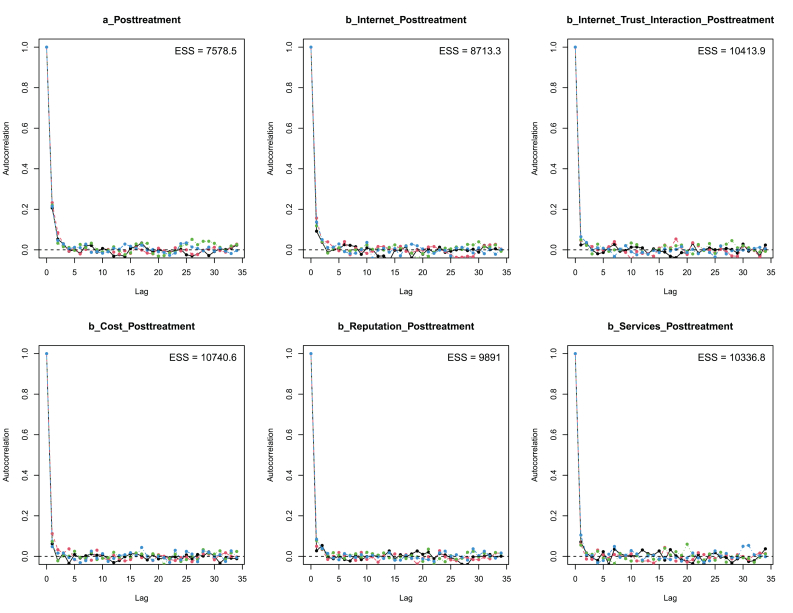
Model 3's autocorrelation plots.

All the coefficients' effective sample sizes obtain relatively large numbers of independent iterations, and Gelman values equal 1 (see Table 4). These are good signals of Model 3's Markov chain convergence. The trace plots (see Figure 16), Gelman plots (see Figure 17), and autocorrelation plots (see Figure 18) again validate the convergence signals.

**Table 4:** Model 3's simulated posteriors.

Parameters	Uninformative prior		Prior-tweaking (belief on effect)		Prior-tweaking (disbelief on effect)		n_eff∗	Rhat∗
	Mean	SD	Mean	SD	Mean	SD		
*Constant*	-0.62	0.31	-0.62	0.30	-0.62	0.31	7402	1
*Internet*	-0.23	0.09	-0.24	0.09	-0.23	0.09	8652	1
*Internet∗Internet_Trust*	0.26	0.05	0.27	0.05	0.25	0.05	10215	1
*Cost*	0.11	0.18	0.11	0.18	0.11	0.18	10314	1
*Reputation*	0.61	0.16	0.61	0.15	0.60	0.16	9452	1
*Services*	-0.63	0.20	-0.63	0.20	-0.63	0.20	10332	1

∗n_eff and Rhat values presented in the tables are the effective sample sizes and Gelman values taken from the simulated results using uninformative prior.

**Figure 19:**
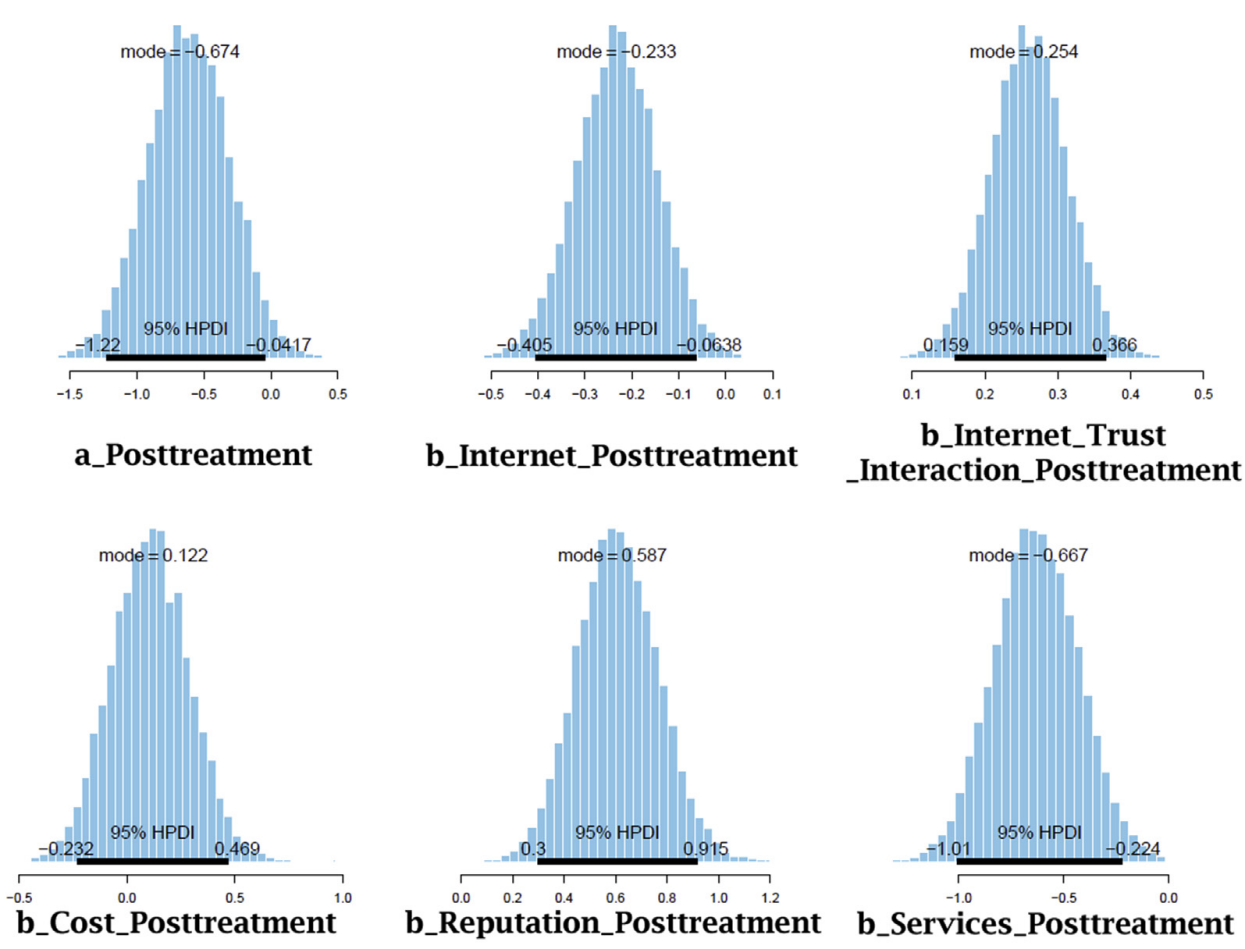
Distributions of Model 3's coefficients with HPDI 95%.

The simulated results of *Internet* and *Internet∗Internet_Trust* using Model 3 remain almost similar to the results acquired using Model 2 ($\pi_{Internet}$ = -0.23 and $\sigma_{Internet}$ = 0.09; $\pi_{Internet*Internet\_Trust}$ = 0.26 and $\sigma_{Internet*Internet\_Trust}$ = 0.05). The probability distributions shown in Figure 19 hint at the high reliability of *Internet*'s and *Internet∗Internet_Trust*'s impacts on *ex-post* assessment of healthcare providers. In particular, the 95% Highest Posterior Distribution Intervals (HPDIs) of *Internet* and *Internet∗Internet_Trust* are entirely on the negative and positive sides, respectively. Moreover, results obtained after the prior-tweaking show that the model is not sensitive to prior belief modification.

Therefore, it is plausible to conclude that the negative association between *Internet* and *Posttreatment* and the positive association between *Internet∗Internet_Trust* and *Posttreatment* are highly reliable and robust.

The simulated results also delineate that *Cost* and *Reputation* positively influence the *ex-post* assessment of providers ($\pi_{Cost}$ = 0.11 and $\sigma_{Cost}$ = 0.18; $\pi_{Reputation}$ = 0.61 and $\sigma_{Reputation}$ = 0.16). Nevertheless, while the association between *Reputation* and *Posttreatment* is highly reliable because its HPDI is entirely located on the positive side, the association between *Cost* and *Posttreatment* only has low confidence because a large proportion of the HDPI still lies on the negative side, and its standard deviation is large. In contrast, *Services* negatively influences the *ex-post* assessment ($\pi_{Services}$ = -0.63 and $\sigma_{Services}$ = 0.20); its negative effect is highly reliable because all the HPDI appears on the negative side.

## 4 Discussion

The current study explored patients’ psychological process of Internet information for decision making and *ex-post* evaluation of their healthcare providers. It is one of the first studies to implement the BMF approach in studying healthcare management. Specifically, the mindsponge mechanism was employed to theoretically construct three models, while Bayesian statistics was performed using a dataset of 1,459 Vietnamese patients to analyze these models.

Model 1's result shows that the information accessibility of Internet sources is positively associated with patients' perceived information sufficiency for choosing a healthcare provider, and trust toward Internet sources facilitates this positive effect. This result is rather straightforward and intuitively expected as it is consistent with former studies on the role of Internet sources of healthcare information (Lemire et al., 2008; Rains, 2007; Sheng and Simpson, 2015). Focusing more on the effect of trust, we again show how trust functions in terms of information processing, which can be considered a “priority pass” within the mindsponge mechanism. This important aspect of trust, especially regarding evaluating an information source before information reception, was investigated in former studies employing the mindsponge framework (Nguyen et al., 2021; Vuong et al., 2021b).

However, our findings also show a rather interesting effect: higher accessibility to Internet information predicts negative *ex-post* evaluation (see results of Model 2 and 3), but this effect is negated by trust in Internet sources. The *ex-post* evaluation is made based on comparing previously acquired information (Internet and non-Internet information) and information acquired from real experience (the treatment). The result of Model 1 suggests that the dependence on Internet information in making decisions may increase together with the amount of information acquired from Internet sources, so it is plausible to say that the negative outcome happens when the information acquired from the Internet is not aligned with the patient's real experience. In other words, a patient might be more likely to consider his/her selection of providers based on Internet information as “worse” than (not as “optimal” as) experienced reality or perception about other providers suggested by non-Internet sources. Following the scarcity principle, perceived low-prevalent characteristics are evaluated more extremely (Ditto and Jemmott, 1989). This means that the perceived high abundance of Internet information may lead to a more “moderate” evaluation, in turn leaving more room for yet-to-be-identified better (more “optimal”) alternatives. Regardless of the causes, this negative relationship holds the dangerous risk of eroding trust toward Internet sources regarding healthcare information.

Trust toward Internet sources was found to negate the feeling of “bad choice” in patients having gone through treatment. It might result from the fact that trust increases the speed of absorbing information from the Internet, which competes with the amount of information received from non-Internet sources. Furthermore, according to mindsponge principles, trust is not naturally given but rather the result of previous evaluation, meaning that those who trust Internet sources have already well-assessed these sources of information. Making a choice is a complex psychological process involving a great deal of information filtering (Vuong and Napier, 2014). Thus, thanks to previous evaluation, patients might know better whether the searched information on the Internet is “good” or “bad” and consequently can make more accurate decisions than those with low trust in their sources.

Additionally, information on the Internet often does not accurately reflect reality, like the case of over-promising advertisements, which cause false expectations in consumers. Overall, the dissimilarities between real experience and Internet information might make patients feel negative about their choices. This emphasizes the importance of quality control and public trust-building for Internet-based healthcare information (Jabeen et al., 2018; Lu et al., 2018; Tonsaker et al., 2014).

Regarding how the perception of important factors leading to provider choice influences post-treatment assessment, we found that cost does not have a significant effect. In contrast, professional reputation has a positive effect, and service has a negative effect. These results show that patients who think the professional reputation of the healthcare providers is the decisive factor in their choice will be more likely to assess that their decision has been optimal after the treatment. The opposite happens for those who have based their choice more on the provider's services. Considering that Vietnamese tend to have a high level of trust toward a small circle of closely known people (An and Phuong, 2021), this pattern of social trust increases the perceived value of information from person-to-person communication. As reputation is the socially established general trust toward a healthcare provider (Shore, 2005), people use this when exchanging information, especially in the popular form of personal recommendation. It can also be speculated that such over-reliance on reputation from close-group channels may lead to biased *ex-post* evaluation (favoring groups' assessment over one's assessment). Regarding people who emphasize the importance of healthcare services in their decision, the results might indicate that, regardless of reasons, the services they received tend to be (perceived as) worse than what they had expected. This aspect points to the role of transparency and reliability in providing healthcare information to the public.

Developing and managing a good digital healthcare system is complex (Mathews et al., 2019). In developing a good digital government-based healthcare system in Vietnam, trust and transparency are among the most crucial factors (Nguyen et al., 2020). Indeed, effective communication through Internet channels requires public trust. Suppose we solely focus on improving information availability in technical aspects (e.g., infrastructure and technology) without paying enough attention to social aspects, particularly the trust factor. In that case, the system will hold the risk of not being used effectively no matter how accessible it may become. Trust-building is timely and requires a systematic approach. The mindsponge mechanism suggests that the human mind has an updating manner, meaning that the evaluation of new information will be based on previous evaluations, creating reinforcing loops. Thus, trust must be built step-by-step, and bad implementations causing serious public distrust would disrupt andreset the effort. Still, they may also reignite and reinforce former negative attitudes.

As our findings highlight the importance of trust and the adverse effects of flooding information on the Internet, an Internet information monitoring framework should be designed and implemented to improve communication effectiveness and eventually facilitate the adoption of the digital health system. Capitalizing on the positive effect of professional reputation on patients’ perceptions, reputational healthcare providers should develop appropriate online resources for patients and promote open discussion between doctors and patients about online health information (Tonsaker et al., 2014).

The current study's limitations are presented and discussed for transparency (Vuong, 2020). The data we use for analysis was collected during 2015–2016. The general public during the COVID-19 pandemic may exhibit some differences in their attitudes toward the healthcare system. Additionally, a 5-year period in our world of rapidly advancing technology can cause significant changes regarding the technical capacity of the digital healthcare system. However, collecting new data using the same methodology during this crisis would face numerous obstacles due to social distancing policies. Our theoretical design and methodology are based on a framework of information processing mechanism (mindsponge mechanism), so they can be easily replicated using new data. Our data were collected in Northern Vietnam, so the found effects might, to some extent, be influenced by the socio-cultural differences among regions. Nevertheless, the findings can still be representative of Vietnamese patients because the regional distinctions are not significant.

## Declarations

### Author contribution statement

Minh-Hoang Nguyen: Conceived and designed the experiments; Performed the experiments; Analyzed and interpreted the data; Contributed reagents, materials, analysis tools or data; Wrote the paper.

Quan-Hoang Vuong: Conceived and designed the experiments; Contributed reagents, materials, analysis tools or data.

Tam-Tri Le: Conceived and designed the experiments; Analyzed and interpreted the data; Wrote the paper.

Viet-Phuong La: Performed the experiments; Contributed reagents, materials, analysis tools or data.

### Funding statement

This research did not receive any specific grant from funding agencies in the public, commercial, or not-for-profit sectors.

### Data availability statement

Data associated with this study has been deposited at https://www.sciencedirect.com/science/article/pii/S2352340916302803?via%3Dihub.

### Declaration of interests statement

The authors declare no conflict of interest.

### Additional information

No additional information is available for this paper.
